# Transferrin Modified GSH Sensitive Hyaluronic Acid Derivative Micelle to Deliver HSP90 Inhibitors to Enhance the Therapeutic Efficacy of Brain Cancers

**DOI:** 10.3390/cancers13102375

**Published:** 2021-05-14

**Authors:** Tilahun Ayane Debele, Ping-Ching Wu, Yu-Feng Wei, Jian-Ying Chuang, Kwang-Yu Chang, Jui-Hung Tsai, Wen-Pin Su

**Affiliations:** 1 Institute of Clinical Medicine, College of Medicine, National Cheng Kung University, No. 35, Tainan 704, Taiwan; z10803012@ncku.edu.tw or; 2 Department of Biomedical Engineering, National Cheng Kung University, Tainan 701, Taiwan; wbcxyz@bme.ncku.edu.tw; 3 Center of Applied Nanomedicine, National Cheng Kung University, Tainan 704, Taiwan; 4 Department of Stomatology, Institute of Oral Medicine, National Cheng Kung University Hospital, College of Medicine, National Cheng Kung University, Tainan 701, Taiwan; 5 Medical Device Innovation Center, Taiwan Innovation Center of Medical Devices and Technology, National Cheng Kung University Hospital, National Cheng Kung University, Tainan 701, Taiwan; 6 Department of Internal Medicine, School of Medicine for International Students, College of Medicine, E-Da Cancer Hospital, I-Shou University, Kaohsiung 824, Taiwan; ed102746@edah.org.tw; 7 The Ph.D. Program for Neural Regenerative Medicine, Taipei Medical University, Taipei 115, Taiwan; chuangcy@tmu.edu.tw; 8 National Institute of Cancer Research, National Health Research Institute, Tainan 704, Taiwan; kwang2@nhri.edu.tw; 9 Departments of Oncology and Internal Medicine, National Cheng Kung University Hospital, College of Medicine, National Cheng Kung University, Tainan 701, Taiwan; n100128@mail.hosp.ncku.edu.tw

**Keywords:** hyaluronic acid, poly(lactic-co-glycolic) acid, GSH-sensitive micelle, brain cancer

## Abstract

**Simple Summary:**

Heat shock protein 90 (HSP90) is a key element of a multi-chaperone complex involved in the stabilizing of many client proteins, oncoproteins, which play essential roles in tumorigenesis. As the result, HSP90 has been taken as a promising target for anticancer therapies. AUY922 has good antitumor activity by inhibiting the ATPase activity of HSP90, while it has certain limitations, including poor water solubility and lack of selectivity, which have incited the development of a novel targeted nanoformulation. In this study, we have successfully synthesized and characterized a GSH-sensitive micelle that can encapsulate hydrophobic AUY922 in its core region to enhance its therapeutic efficacy against brain cancers. All in vitro and in vivo experimental results showed nanoformulated AUY922 has a better therapeutic efficacy against brain cancer in comparison to the free AUY922.

**Abstract:**

Herein, GSH-sensitive hyaluronic acid-poly(lactic-co-glycolic acid) (HA-SS-PLGA) was synthesized. Surface modification of PLGA with hyaluronic acid produced a highly stable micelle at physiological pH while a micelle was destabilized at a higher GSH level. Fluorescence microscopy results showed that rhodamine-encapsulated micelle was taken up by brain cancer cells, while competitive inhibition was observed in the presence of free HA and free transferrin. In vitro cytotoxicity results revealed that transferrin-targeted nanoformulated AUY922 (TF-NP-AUY922) shows higher cytotoxicity than either free AUY922 or non-targeted AUY922-loaded micelles (NP-AUY922). In comparison to the control groups, free AUY922, TF-NP-AUY922 or NP-AUY922 treatment revealed the upregulation of HSP70, while the expression of HSP90 client proteins was simultaneously depleted. In addition, the treatment group induced caspase-dependent PARP cleavage and the upregulation of p53 expression, which plays a key role in apoptosis of brain cancer cells. In vivo and ex vivo biodistribution studies showed that cypate-loaded micelle was taken up and accumulated in the tumor regions. Furthermore, in vivo therapeutic efficacy studies revealed that the AUY922-loaded micelle significantly suppressed tumor growth in comparison to the free AUY922, or control groups using tumor-bearing NOD-SCID mice. Moreover, biochemical index and histological analysis revealed synthesized micelle does not show any significant cytotoxicity to the selected major organs. Overall, a synthesized micelle is the best carrier for AUY922 to enhance the therapeutic efficiency of brain cancer.

## 1. Introduction

Glioblastoma multiform (GBM) is the most common primary malignant brain tumor and accounts for more than 60% of all brain tumors in adults [1,2,3]. Currently, several treatment methods and therapeutic agents have been discovered to treat GBM [4,5,6]. Of these, temozolomide (TMZ) showed better antitumor activity and fewer side effects. However, its survival benefit remains unsatisfactory due to the rapid occurrence of resistance and tumor relapse [7,8,9]. To overwhelm TMZ-related resistance, the identification of molecular targets that control multiple signaling pathways is highly required. As a result, heat shock protein 90 (HSP90) has become a potential therapeutic target for the identification and development of a new generation of anticancer drugs to treat different forms of cancers, including GBM [10,11]. A different study showed heat shock proteins are overexpressed in a wide range of cancers and play a vital role in the folding and function of a large number of client proteins, oncoproteins, which are involved in tumor cell proliferation, differentiation, invasion, and metastasis [12,13,14,15,16]. Currently, several small molecules such as geldanamycin, tanespimycin, alvespimycin, retaspimycin, AUY922, and novobiocin, are being highly explored to selectively inhibit HSP90 and to control multi oncogenic signals [16,17,18,19]. AUY922 (luminespib), a potent second-generation HSP90 inhibitor, has good antitumor effects by inhibiting the ATPase activity of HSP90 [20,21,22]. In addition, AUY922 has shown nanomolar efficacy against a wide range of human cancer cells in vitro and inhibits the progression of a variety of tumors in vivo [23,24,25]. However, AUY922 has several limitations, due to its hydrophobicity (water insolubility) and lack of selectivity, which have incited the development of a novel targeted nanoformulation.

In the past few decades, several polymer-based nanoparticles (NPs) have been synthesized to enhance the therapeutic efficacy of anticancer drugs [26,27]. Even though traditional polymeric NPs could improve pharmacodynamics and pharmacokinetics of the therapeutic agents, the synthesis of targeted stimuli nanocarriers is required to enhance effective therapeutic outcomes by minimizing side effects of untargeted normal cells and by controlling premature drug release. As a result, the synthesis of ligand-targeted polymeric NPs, which can be activated by tumor associated-stimuli such as pH, GSH, ROS and specific enzymes which are primarily overexpressed in cancer cells, have gained great attention [28]. Of these stimuli, responsive, cleavable crosslinking of the NPs either at the shell or core regions has received increasing attention to maintain the stability and to prevent premature drug release. The intracellular GSH levels of the cancer cell are four times higher than the normal cells’ intracellular environments [29,30,31,32].

In this study, a transferrin-targeted GSH-sensitive micelle was synthesized using hyaluronic acid as the backbone and as a ligand. Hyaluronic acid (HA) is a naturally occurring glycosaminoglycans (GAGs) with a poly repeating disaccharide, β-d-*N*-acetyl glucosamine, and β-d-glucuronic acid, via alternative linkage of β- 1→3 and 1→4 [33]. Due to its versatile biological properties along with its biocompatibility, biodegradability, and its intrinsic targeting ability, HA has become the best candidate polymer in drug delivery systems [34,35].

In the drug delivery system, one of the strategies to enhance the therapeutic index of the anticancer drug is to selectively deliver to the site of interest, thus avoiding unwanted cytotoxicity to healthy cells using specific ligands against the surface receptor, which are primarily expressed on cancer cells rather than the normal cells. The transferrin receptor is a cell membrane-associated glycoprotein that serves as a gatekeeper in regulating the cellular uptake of iron from transferrin protein [36]. In addition, transferrin protein has received major attention in drug targeting to enhance an efficient cellular uptake of transferrin (TF)-modified nanoformulated drugs due to the high expression of transferrin receptors on the surface of cancer cells [37]. Moreover, CD44, a non-kinase transmembrane receptor that binds HA, is a widely expressed adhesion molecule in a variety of tumor types, including brain cancer cells [38]. Thus, dual targeting (such as HA and transferrin) can improve the therapeutic efficacy of anticancer drugs by enhancing cellular uptake and accumulation at the site of interest.

Herein, hyaluronic acid was conjugated with PLGA-cystamine to form HA-SS-PLGA via EDC/NHS chemistry to extend stability in the circulation due to disulfide crosslinking, high drug loading, and fast drug release due to excess GSH after cellular uptake by cancer cells via a receptor (both transferrin and CD44 receptor)-mediated endocytosis. To the best of our knowledge, this is the first study on transferrin-targeted AUY922 nanoformulation to treat brain cancer.

The detailed chemical properties of the synthesized co-polymer (HA-SS-PLGA) and its precursors were analyzed using ^1^H-NMR and FTIR. The properties of the micelle, such as morphology, particle size, critical micelle concentration, encapsulation efficiency (%), drug loading capacity (%), and in vitro drug release behavior were evaluated in detail. Cellular uptake of the rhodamine-loaded micelle was observed through fluorescence microscopy, whereas cellular cytotoxicity was investigated by flow cytometry, colony assay, and MTT assays using U87, temozolomide-sensitive (P5), and temozolomide-resistant (P5-TMZ-R) brain cancer cells. The detailed molecular mechanism action of the free AUY922 and AUY922-loaded micelle was studied using Western blotting. Finally, in vivo and ex vivo biodistribution of the cypate-loaded micelle and anti-tumor activity of the AUY922-loaded micelle and free AUY922 were evaluated using U87-bearing NOD-SCID mice.

## 2. Experimental Methods

### 2.1. Materials

Sodium hyaluronate (Mw = 14 kDa) was purchased from Life Core Biomedical (Chaska, MN, USA). Poly(lactic-co-glycolic acid) (PLGA, 35.8 kDa), 1-ethyl-3-(3-dimethylaminopropyl) carbodiimide (EDC), *N*-hydroxysuccinimide (NHS), and cystamine were all purchased from Sigma-Aldrich (St. Louis, MO, USA). AUY922 and temozolomide were purchased from Amersham Biosciences (Piscataway, NJ, USA). Cypate was kindly donated from Professor Ping-Ching Wu’s lab. All other reagents were analytical grade and used without further purification. Distilled water was used throughout the experiments and water used in all the experiments was purified using an Aqua-Max-Ultra water purification system (Younglin Co., Anyang, Korea).

### 2.2. Synthesis and Characterization of GSH Responsive HA-SS-PLGA

**Scheme 1:**
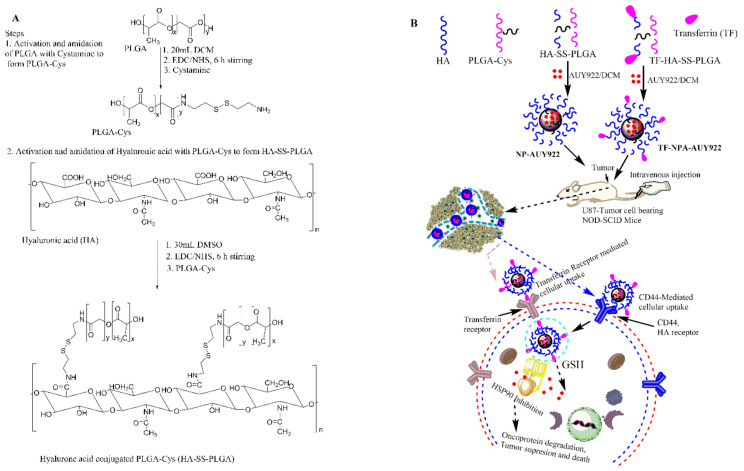
(**A**) Scheme of HA-SS-PLGA synthesis and (**B**) transferrin and CD44 receptor-mediated cellular uptake of AUY922-loaded micelle.

The GSH-sensitive hyaluronic acid derivative (HA-SS-PLGA) was synthesized using two separate reaction steps, as shown in Scheme 1. First, the carboxyl group of PLGA was activated with EDC/NHS and then one of the amine groups of cystamine reacted with the activated carboxyl group of PLGA to form PLGA-Cys (detailed synthesis protocols were mentioned in our previous paper) [39]. In the second reaction step, the carboxyl group of HA was activated in the presence of EDC/NHS and conjugated with the PLGA-Cys to form HA-SS-PLGA copolymer. Briefly, HA (0.05 g, 0.132 mmol) was dissolved in anhydrous DMSO (15 mL) and then activated by EDC (0.05 g, 0.264 mmol) and NHS (0.03 g, 0.264 mmol) for 6 h under stirring. Then, PLGA-Cys (1 g, ~0.025 mmol) (5:1 mol/mol ratio) was added into the above reaction mixture in the presence of 40 μL of TEA (1.95 mmol) and the reaction continued for 24 h under stirring, at room temperature. Then, the reaction mixture was purified for 96 h against distilled water using a dialysis bag (spectra/Por, MWCO: 12–14 kDa), within 6 h interval water exchange. Finally, the HA-SS-PLGA solution was freeze-dried, stored at 4 °C and used for further experiments. ^1^H-NMR and FTIR was used to confirm PLGA-Cys and HA-SS-PLGA formation. The infrared absorption spectra were collected from 4000–620 cm^−1^ using a Perkin Elmer spectrometer. For ^1^H-NMR analysis, the precursors (i.e., HA and cystamine) were dissolved in D_2_O, whereas PLGA was dissolved in DMSO-d6 and products (PLGA-Cys and HA-SS-PLGA) were dissolved in DMSO-d6 and analyzed by a Bruker AVANCE 600 MHz NMR spectrometer (Billerica, MA, USA).

### 2.3. Transferrin-Conjugated HA-SS-PLGA (TF-HA-SS-PLGA) Synthesis

Transferrin (TF) was conjugated to the HA-SS-PLGA using the EDC/NHS coupling reaction. Briefly, HA-SS-PLGA (320 mg, ~0.00384 mmol, by taking into consideration ~40 of HA carboxyl group was conjugated with PLGA-Cys, from ^1^H-NMR results) was dissolved in 10 mL of PBS buffers and 5 mL of DMSO and activated in the presence of excess EDC and NHS at room temperature for 6 h with gentle stirring. Then, 10 times less molar ratio of TF (32 mg) was dissolved in 5 mL of PBS solution and mixed with NHS-activated HA-SS-PLGA solution, and reacted with gentle stirring at room temperature for 24 h. After the reaction, the resulting solution was dialyzed using the dialysis membrane (molecular weight cut off, MWCO 12–14 kDa) against distilled water to remove the unreacted reagents for 96 h within 12 h of distilled water exchange. Finally, the powder of TF-HA-SS-PLGA was obtained by lyophilization and stored at 4 °C until it was used for the other experiments.

### 2.4. Micelle Synthesis and AUY922 Loading

The micelle containing drugs was synthesized using the emulsion solvent evaporation method [39,40]. Briefly, HA-SS-PLGA and/or TF-HA-SS-PLGA (20 mg each) and AUY922 (10 mg) were dissolved in dichloromethane (10 mL) under stirring until a clear homogenous solution was obtained. Next, the above solution was added into 10 mL of 1% polyvinyl alcohol (PVA) under stirring, followed by emulsification using a probe-type sonicator at 60% amp, (pulse 30 on and 15 off) for 5 min. Then, the DCM was removed at room temperature, followed by centrifugation at 13,000 rpm for 20 min (repeated 3×) to remove unloaded AUY922. Finally, the drug-loaded micelle pellets were resuspended in distilled water or phosphate buffered saline (PBS) and incubated at 4 °C for further in vitro and in vivo experiments. The empty micelle was similarly prepared using HA-SS-PLGA and/or TF-HA-SS-PLGA without adding drugs. Moreover, rhodamine B or cypate was encapsulated in the HA-SS-PLGA or TF-HA-SS-PLGA within the same protocols by replacing rhodamine b or cypate instead of AUY922. Dynamic light scattering (Malvern Zetasizer Nano S) and TEM (with a JEOL JEM-100CX-II instrument) were used to determine particle size and morphology of the synthesized micelle. Furthermore, the AUY922 encapsulation and loading ability of micelle was measured using UV–VIS spectroscopy at 310 nm wavelength. Encapsulation efficiency (EE) and drug loading capacity (DL) was calculated by using the following equation:

$$\mathrm{EE}\;\left(\%\right)=\frac{\mathrm{Weight}\;\mathrm{of}\;\mathrm{AUY}922\;\mathrm{in}\;\mathrm{the}\;\mathrm{micelle}}{\mathrm{Weight}\;\mathrm{of}\;\mathrm{AUY}922\;\mathrm{initial}\;\mathrm{feeding}\;}\times 100\;\mathrm{DL}\;\left(\%\right)=\frac{\mathrm{Weight}\;\mathrm{of}\;\mathrm{AUY}922\;\mathrm{in}\;\mathrm{the}\;\mathrm{micelle}}{\mathrm{Weight}\;\mathrm{of}\;\mathrm{drug}-\mathrm{loaded}\;\mathrm{micelle}}\times 100$$

### 2.5. In Vitro Drug Release

The in vitro AUY922 release from micelle was assessed using dialysis methods. Briefly, 2 mL of AUY922-loaded micelle solution was taken and the amount of AUY922 released was monitored using a dialysis bag (6–8 kDa) against 10 mL of PBS solution (pH 7.4) in the presence and absence of 5 mM GSH at 37 °C. At a predetermined time, 1 mL of releasing medium was taken, and the same volume was added back to keep the volume constant. The absorbance of AUY922 was measured at 310 nm wavelength and the concentration of AUY922 was calculated based on the pre-established calibration curve. Results were plotted as the cumulative release (%) against time.

### 2.6. In Vitro Stability

The in vitro stability of the empty micelle and AUY922-loaded micelle were investigated in protein solutions [31,41]. Briefly, empty and AUY922-loaded micelle solution was mixed with 10% FBS solution and change in the particle size was monitored using DLS.

### 2.7. In Vitro Cytotoxicity

The in vitro cytotoxicity of PLGA-Cys, HA-SS-PLGA, AUY922, and AUY922-loaded micelles were evaluated against human brain cancer cells, U87, P5, and P5-TMZ-R, using MTT assay. The detailed culture protocols and the source of human brain cancer cells are briefly discussed in the Supplementary Materials. Briefly, 5 × 10^3^ U87, P5, and P5-TMZ-R brain cancer cells were seeded for 24 h in 96-well plates. Then, serial dilution of PLGA-Cys and HA-SS-PLGA (500, 250, 125, 62.5, and 31.25 µg/mL), free AUY922 and AUY922-loaded micelles (at an AUY922 concentration of 100, 50, 20, 10, and 5 nM) were added and incubated for 72 h at 37 °C in 5% CO_2_. After 72 h of incubation, 20 μL of MTT (5 mg/mL) solution was added and further incubated for 3–4 h. The medium was removed and 100 μL of DMSO was added to dissolve formazan crystals. The absorbance was read at the test wavelength of 492 nm and the cell viability (%) was calculated as:

$$Cell\;viability\left(\%\right)=\frac{absorbance\;of\;test\;cells-absorbance\;of\;reference}{absorbance\;of\;controlled\;cells-absorbance\;of\;reference}\times 100$$

### 2.8. Colony Assay

Colony assay was determined after treated U87, P5 and P5-TMZ-R brain cancer cells using a serial dilution of free AUY922 and AUY922-loaded micelles (at AUY922 dosage of 0.5, 1, 2, 4, and 8 nM). After 2 weeks of treatment, the medium was removed, washed with PBS, and treated with methanol to fix a cell for 2 h. Next, methanol was removed, washed with PBS, and 0.5% crystal violet solution was added overnight. Then, the plate was washed under running tap waters until the background was clear and then air-dried at room temperature. Finally, a Microtek scanner was used to scan each plate.

### 2.9. Apoptosis Assay

The Annexin V-Alexa Fluor 488 apoptosis detection kit was used to quantify apoptotic and necrotic cells by standard fluorescent activated cell sorting (FACS) assay. Briefly, 1 × 10^5^ U87, P5, and P5-TMZ-R brain cancer cells were seeded into 6-well plates for 24 h. Then, free AUY922 and AUY922-loaded micelles (at an AUY922 concentration of 100 nM) were added and incubated with cells for 72 h at 37 °C. The cells were then washed, collected, and resuspended in 1× annexin-binding buffer. Alexa Fluor^®^ 488 annexin V and propidium iodide (PI) was added according to the manufacturer’s recommendation. Samples were incubated in the dark for 15 min at room temperature. An additional 400 μL of 1× annexin-binding buffer was added and mixed gently with the samples before analysis.

### 2.10. Cell Cycle Analysis

U87, P5, and P5-TMZ-R brain cancer cells were seeded in a 6-well plate at a cell density of 1 × 10^5^ overnight at 37 °C. After 24 h of incubation, free AUY922 and AUY922-loaded micelles (at an AUY922 concentration of 100 nM) were added and incubated for 48 h. The cells were then washed with PBS, collected, and fixed using ice-cold ethanol overnight. Finally, the cell suspension was centrifuged at 1000 rpm for 5 min, washed with PBS, and stained with PI/Triton X-100/RNA_ase_ solution for 30 min at room temperature before analysis using a BD FACS Calibur flow cytometer.

### 2.11. Cellular Uptake and Competitive Inhibition Study

Cellular uptake and competitive inhibition of the rhodamine-loaded micelle was investigated using P5 and P5-TMZ-R brain cancer cells via fluorescence microscopy [32]. Briefly, 2.2 × 10^4^ P5 and P5-TMZ-R brain cancer cells were seeded in the four or six-well microslide chamber overnight at 37 °C. Then, each cell was treated with rhodamine-loaded micelles at a rhodamine concentration of 1 μg/mL, (300 ng per well) and incubated for 3 h at 37 °C. Cellular uptake and the competitive inhibition of transferrin-targeted rhodamine-loaded micelle were investigated using fluorescence microscopy after incubating for 3 h at 37 °C. For competitive inhibition, the cell was incubated with free transferrin (5 mg/mL) and hyaluronic acid (5 mg/mL) for 1 h before adding the rhodamine-loaded micelle at 4 °C. Similarly, cellular uptake and competitive inhibition were investigated using flow cytometry.

### 2.12. Western Blot Analysis

The protein expression was assessed using Western blotting analysis using P5, P5-TMZ-R, and U87 brain cancer cells. Briefly, P5, P5-TMZ-R, and U87 brain cancer cells were treated with free AUY922 and nanoformulated AUY922 (at an AUY922 concentration of 100 nM) for 48 h. After 48 h of incubation, cultured cells were washed with PBS, trypsinized, and lysed in RIPA buffer supplemented with protease inhibitor. The protein concentrations were evaluated with a Bio-Rad protein assay dye reagent concentrate (Cat. #5000006, Pierce™, Thermo Scientific, Waltham, MA, USA). The samples were heated at 95 °C for 10 min to denature the proteins, separated on 8–12% SDS-polyacrylamide gel, and electrophoretically transferred to polyvinylidene fluoride (PVDF) membrane (Millipore Corporation, Billerica, MA, USA). Membranes were blocked with 5% milk powder in Tris-buffered saline containing 0.05% Tween (1×-TBST buffer) for 1 h at room temperature, after which primary antibodies were added and incubated overnight at 4 °C. The membranes were subsequently washed in TBST (3×), after which secondary horseradish peroxidase-conjugated antibodies were added in 5% milk powder in TBST and incubated for 1 h at room temperature. After extensive washing in TBST, antibody binding was detected after adding ECL Western blotting substrate (Western lightning Plus ECL, Pierce™, Thermo Scientific, Waltham, MA, USA) and the imaging blot was taken using the Konica Minolta SRX-101A medical film processor. The immunoblotting was performed with the following antibodies: HSP90 (cell signaling, #4874), HSP70 (cell signaling, #4872), β-Actin (cell signaling, #3700, Boston, MA, USA), Caspase 3 (cell signaling, #14220), PARP (cell signaling, #9542), p53 (cell signaling, #9284), AKT (cell signaling, #9272), pAKT (cell signaling, Ser473, #9271), and ERK2 (cell signaling, #9102).

### 2.13. In Vivo and Ex Vivo Biodistribution Study

The NOD-SCID (6–8 weeks of age, the weight of 20–25 g) mice were obtained from the College of Medicine, National Cheng Kung University, Tainan, Taiwan. The ethical committee of an animal experiment of National Cheng Kung University approved the experiment protocol. Briefly, 1 × 10^7^ U87 brain cancer cells suspended in Matrigel were subcutaneously seeded by inoculation in the back armpit of the NOD-SCID mice. After two weeks of tumor growth (~100 mm^3^), cypate-encapsulated micelle (1 mg/kg weight, 100 μL) was intravenously (i.v.) injected into the tail vein of tumor-bearing mice. The in vivo fluorescent scans were performed by using the IVIS imaging system at 0, 1, 3, 6, and 24 h of post-injection. Similarly, ex vivo fluorescent scans were performed by sacrificing mice after 24 h post-injection.

### 2.14. In Vivo Anti-Tumor Efficacy Study and Biochemical Index Analysis

In vivo anti-tumor efficacy of the free AUY922 and AUY922-loaded micelle was investigated after two weeks of tumor growth. Briefly, PBS (control), NP-AUY922, and TF-NP-AUY922 were injected via the lateral tail vein of NOD-SCID tumor-bearing mice (at the AUY922 concentration of 20 mg/kg of mice), whereas, free AUY922 was injected intraperitoneally (i.p.) to NOD-SCID tumor-bearing mice at a dosage of 40 mg/kg weight of mice for two weeks (twice per week for a total of five injections). The change in tumor volume was measured twice per week using a vernier caliper and volume was calculated as V = a × b^2^ × 0.5, where a and b indicate the major and minor axis of a tumor, respectively. Furthermore, the blood sample of NOD-SCID mice was collected and centrifuged at 3000 rpm for 10 min to separate serum and blood cells. Automated clinical chemistry analyzer (Fuji DRI-CHEM 4000i) was used to measure glutamate pyruvate transaminase (GPT) and creatinine (CRE) concentration to evaluate liver and kidney functional tests, respectively.

### 2.15. Statistical Analysis

All the experiments were carried out in or over triplicate and a one-tailed student’s t-test was performed to assess the statistical significance of the results (* *p* < 0.05, ** *p* < 0.01, and *** *p* < 0.001).

## 3. Results and Discussion

### 3.1. Synthesis and Characterization of PLGA-Cys and HA-SS-PLGA Conjugates

The HA-SS-PLGA copolymer was synthesized by conjugating the carboxyl group of hyaluronic acid (HA) with an amino group of derivatized PLGA (i.e., PLGA-Cys) in the presence of EDC and NHS, as shown in Scheme 1. In this study, HA was used as the backbone of the carriers, as the ligands, or as a shell to enhance stability and water solubility in the circulation, whereas cystamine was used as the GSH-sensitive segments in addition to link PLGA, hydrophobic segment, and HA to facilitate micelle formation to encapsulate hydrophobic HSP90 inhibitor, AUY922. Moreover, transferrin (TF) was used as the ligands to enhance TF-receptor-mediated cellular uptake in addition to HA which could be used to target the CD44 receptor. The structure of PLGA-Cys and HA-SS-PLGA was confirmed using ^1^H-NMR and FTIR. In addition, UV–VIS was used to confirm AUY922 loading and release from the HA-SS-PLGA micelle.

**Figure 1:**
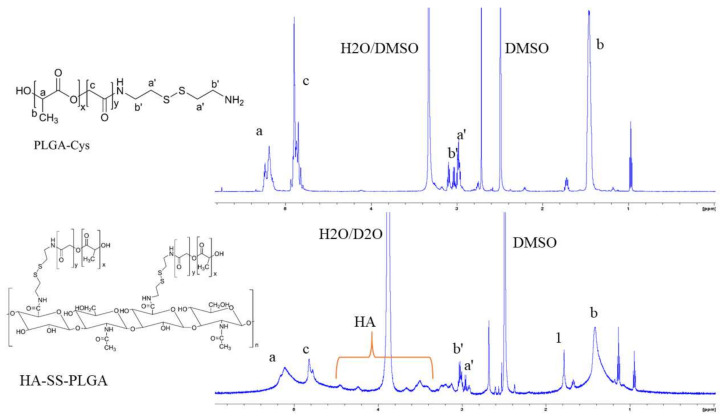
^1^H-NMR of HA-SS-PLGA and PLGA-Cys in DMSO-d6/D2O and DMSO-d6, respectively.

**Figure 2:**
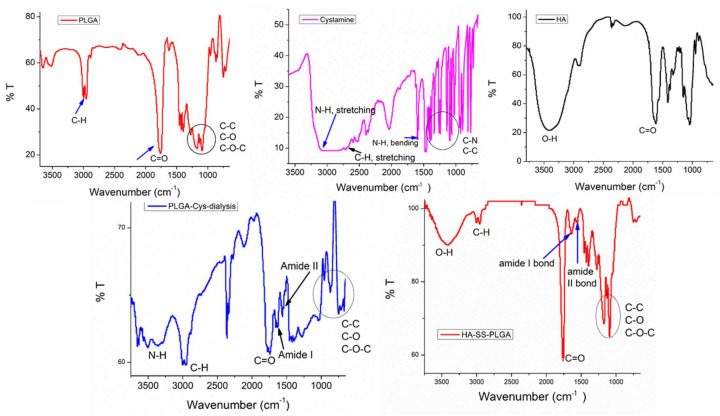
FTIR spectrum of HA-SS-PLGA and its precursors.

As revealed in Figure 1, the ^1^H-NMR peaks were observed at 4.35 ppm (H-1 from d-glucuronic acid) and 4.55 ppm (H-1 from *N*-Acetyl-d-glucosamine) which belongs to the major repeating monosaccharide units of HA. The proton signal of the acetyl groups of HA (CH_3_, –C = O) was found at 1.9 ppm. Furthermore, the other proton of HA disaccharide units (H-2, H-3, H-4, H-5, and H-6) was found at ~3.2–3.9 ppm. After the conjugation of HA with PLGA-Cys, additional peaks were observed at the chemical shifts of 1.48, 4.88, and 5.21 ppm, which correspond to the methyl (3H, –CH_3_), methine (1H, –CH–), and methylene (2H, –OCH_2_–C = O) groups of PLGA, respectively. In addition, the chemical shifts at 2.98 and 3.01 ppm were observed which corresponds to the different methylene (2H, –CH_2_–S–; 2H, –CH_2_–N) groups of the cystamine, the linker of HA with PLGA. Similarly, the chemical structures of the HA-SS-PLGA, PLGA-Cys and its precursors were further confirmed using FTIR as shown in Figure 2. All major characteristic peaks of HA-SS-PLGA, PLGA-Cys, and its precursors were observed within the range of 4000−620 cm^−1^. Several major peaks were observed at ~3200–3364 cm^−1^ (–OH stretching of HA and NH stretching or cystamine), 2883−2915 cm^−1^ (C−H stretching of HA and PLGA), 1756 cm^−1^ (stretching peaks of carbonyl group), 1320–1410 cm^−1^ (C−H bending of O–CH_2_ and C–H bending of CH_3_), and 1021–1075 cm^−1^ (C−C–O stretching from ester). In addition, there is a new peak at 1619 cm^−1^ (amide I bond) and 1538 cm^−1^ (amide II bond) which confirms amide bond formation between PLGA/cystamine and HA/PLGA-Cys. Overall, both ^1^H-NMR and FTIR results confirmed the successful synthesis of PLGA-Cys and HA-SS-PLGA.

### 3.2. Synthesis and Characterization of AUY922-Loaded Micelle

The micelle was formed due to the amphiphilic nature of HA-SS-PLGA (i.e., hydrophilic HA and hydrophobic PLGA) in water. The micelle-forming tendency of HA-SS-PLGA was confirmed by measuring the critical micelle concentration (CMC) using pyrene as a fluorescent probe. As shown in Figure S1, the CMC value of amphiphilic HA-SS-PLGA was determined to be ~0.00758 mg/mL. This low CMC value showed that a synthesized micelle would have good stability, even after extreme dilution by the larger volume of systemic circulation in the body [42,43].

**Figure 3:**
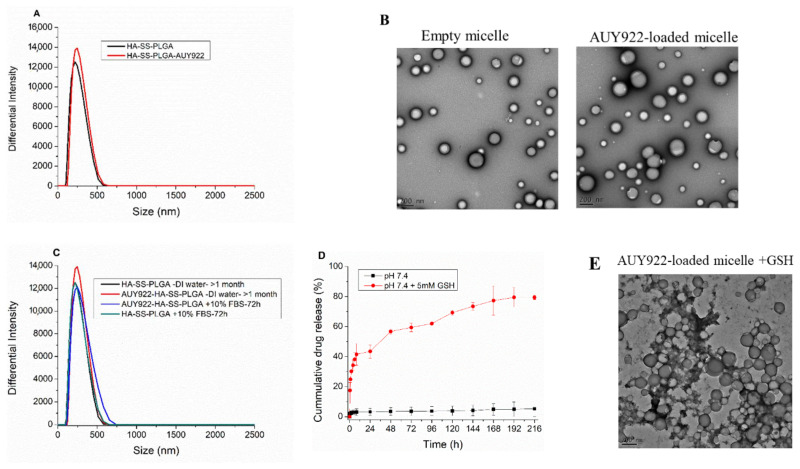
(**A**) Particle size using DLS, (**B**) TEM image of empty micelle and AUY922-loaded micelle, (**C**) Particle size stability in DI water and 10% FBS, (**D**) in vitro drug release in the presence and absence of GSH and (**E**) TEM image of AUY922-loaded micelle in the presence of GSH (5 mM).

After the determination of the CMC value, the drug-loaded micelle was synthesized by encapsulating hydrophobic AUY922 via emulsion solvent evaporation methods. The mean particle size and zeta potential of the AUY922-loaded micelle were 227.27 ± 5.10 and −36.23 ± 1.97 mV, respectively, with a low polydispersity index (PDI; 0.18 ± 0.06), as shown in Figure 3A, based on the DLS measurement. Furthermore, the smooth surface and uniform spherical morphology was observed for the empty micelle and AUY922-loaded micelle using TEM, as shown in Figure 3B. The zeta potential results showed that both the drug-loaded and empty micelle displayed a negative surface charge of approximately −36 mV, which can prolong their circulating half-lives because of the less nonspecific adsorption of plasma proteins. Drug loading (DL %) and encapsulation efficiency (EE %) of the micelle were also important for further clinical application. As per calculated DL% and EE% of the micelles were 10.37% and 87.0%, respectively.

The micelle particle stability is an important factor in the clinical applications of drug delivery systems. The particle size stability of the empty and AUY922-loaded micelle was determined in 10% FBS. As shown in Figure 3C, the particle size of the micelle was not significantly changed after incubation in 10% FBS for 72 h; micelle was stable in DI water for more than 1 month, which revealed a synthesized micelle is stable enough to arrive at tumor sites in blood circulation with less/non-protein adsorption.

Furthermore, in vitro drug release was investigated using PBS buffer (pH 7.4) in the presence and absence of GSH. As revealed in Figure 3D, more AUY922 release was observed in the presence of GSH (5 mM) than at the physiological pH value, which confirmed the synthesized micelle is GSH-sensitive. This result was further supported using both TEM and DLS and the result revealed that GSH induced particle size increment from the DLS result (as summarized in Table S1) and the smooth spherical surface of the micelles was lost, and the micelle was disassembled from TEM images, as shown in Figure 3E.

### 3.3. In Vitro Cytotoxicity Studies

**Figure 4:**
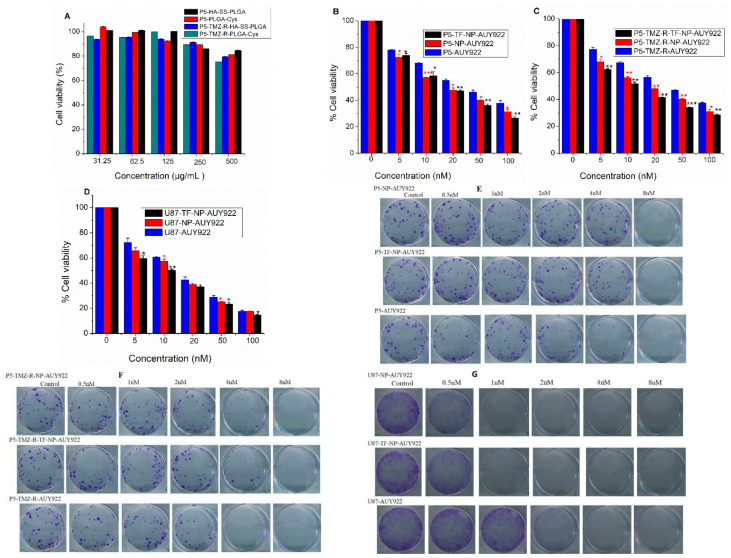
MTT in vitro cytotoxicity results using P5 and P5-TMZ-R cancer cells (**A**) HA-SS-PLGA and PLGA-Cys; (**B**) and (**C**) free AUY922, TF-NP-AUY922, and NP-AUY922, (**D**) free AUY922, TF-NP-AUY922 and NP-AUY922 using U87 brain cancer cells, (**E**–**G**) representative images of the crystal violet-stained colonies using P5, P5-TMZ-R, and U87 brain cancer cells, respectively. (* *p* < 0.05, ** *p* < 0.01, *** *p* < 0.001).

In comparison to free drugs, nanoformulated drugs show different anticancer activity in the in vitro experiments which may be due to the drug release mechanism or their direct interaction with the cell of interest [39]. Hence, to study the in vitro cytotoxicity effect of the AUY922-loaded micelle, free AUY922, PLGA-Cys, and HA-SS-PLGA in the cellular proliferation of cancer cells, cell viability was evaluated by the MTT assay using brain cancer cells (i.e., U87, P5, and P5-TMZ-R). As shown in Figure 4A, PLGA-Cys and HA-SS-PLGA showed less cytotoxicity and had no significant cytotoxicity to both P5 and P5-TMZ-R cancer cells at the maximum tested concentration, which shows synthesized copolymer is biocompatible and can be used for in vivo studies. In addition, as shown in Figure 4B–D, the AUY922-loaded micelle and free AUY922 could inhibit the growth of P5, P5-TMZ-R, and U87 cancer cells in a concentration-dependent manner. TF-NP-AUY922 revealed slightly higher cytotoxicity towards P5, P5-TMZ-R, and U87 cancer cells than free AUY922 and NP-AUY922. This is maybe related to higher expression of the transferrin receptor in the cancer cells which will enhance cellular uptake of the transferrin-targeted AUY922-loaded micelle in addition to the slow drug release mechanism, which will prevent the drugs’ efflux. The half maximal inhibitory concentration (IC50) of free AUY922, NP-AUY922, and TF-NP-AUY922 against P5, P5-TMZ-R, and U87 was summarized in Table 1. Most interestingly, we found that free AUY922 or nanoformulated AUY922 shows better cytotoxicity against P5-TMZ-R in comparison to temozolomide-sensitive (P5) brain cancer cells. This shows HSP90 inhibition is important to reverse temozolomide resistance in brain cancers and which further would enhance the therapeutic efficacy of anti-cancer drugs.

Furthermore, long-term in vitro cytotoxicity effects of free drugs and nanoformulated drugs were evaluated using the colony assay. As shown in Figure 4E–G, free AUY922 and nanoformulated AUY922 significantly inhibited the colony-forming ability of P5, P5-TMZ-R, and U87 brain cancer cells at a low concentration of AUY922. In comparison to the P5 and P5-TMZ-R cancer cells, a low concentration of nanoformulated AUY922 shows better cytotoxicity against U87 brain cancer cells. Together with the MTT assay, this result indicates that nanoformulated or free AUY922 has dose-dependent anti-proliferative actions against brain cancer cells.

### 3.4. Apoptosis and Cell Cycle Analysis

**Figure 5:**
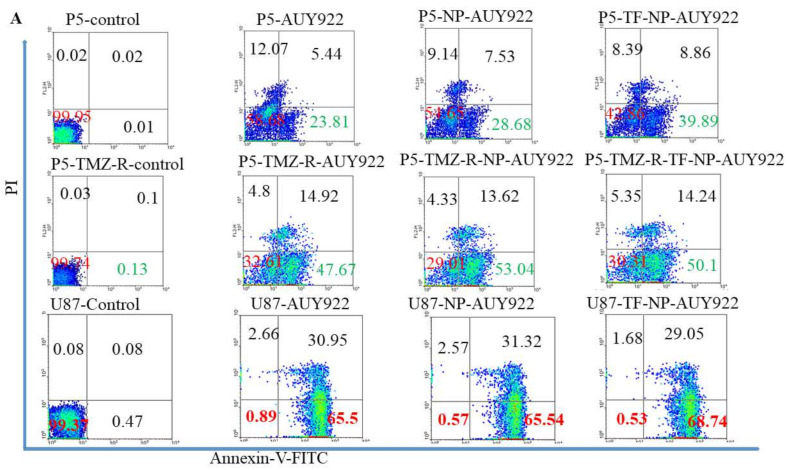

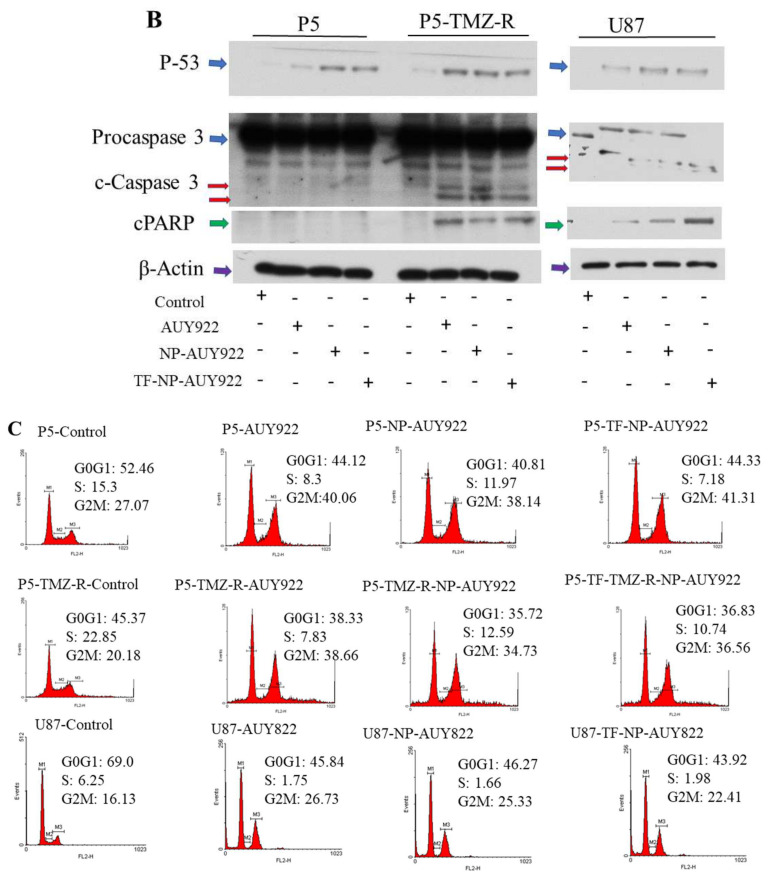
(**A**) Apoptosis assay of free AUY922, NP-AUY922, and TF-NP-AUY922 after 72 h of incubation at a concentration of 100 nM for free or nanoformulated AUY922. (**B**) Expression of P53, activation of caspase 3 (cleaved caspase 3), activation of PARP (cPARP) and β-actin proteins in P5, P5-TMZ-R, and U87 brain cancer cells after treating for 48 h using 100 nM free AUY922, NP-AUY922, and TF-NP-AUY922. (**C**) Cell cycle analysis of free AUY922, NP-AUY922, and TF-NP-AUY922 using P5, P5-TMZ-R, and U87 brain cancer cells after 48 h of incubation at a concentration of 100 nM for free or nanoformulated AUY922. In (**B**), the actin in the P5/P5-TMZ-R part of the experiment is identical as shown in Figure 7A, and the actin in the U87 part of the experiment is identical as shown in Figure 7A,B, because they are part of the same original blots probed with different antibodies of interest. The figures are re-organized as presented to better align with the data demonstrated in the Results section.

Apoptosis is one of the important marks to evaluate the anticancer activity of drugs. The apoptotic effect of free AUY922 and AUY922-loaded micelles against P5, P5-TMZ-R, and U87 brain cancer cells was investigated using an annexin V and PI assay. Free AUY922 and AUY922-loaded micelles induce apoptosis in all cell lines, P5, P5-TMZ-R, and U87 brain cancer cells. As shown in Figure 5A, an increase in the apoptosis of P5, P5-TMZ-R, and U87 brain cancer cells was observed after treatment with free AUY922, AUY922-loaded micelles, and transferrin-targeted AUY922-loaded micelles for 72 h. The percentage of apoptotic cells among those treated with free AUY922 at 100 nM was 29.2%, 62.5%, and 96.45% for P5, P5-TMZ-R, and U87 brain cancer cells, whereas it was 0.03%, 0.23%, and 0.55 in untreated cells, respectively. Similarly, 36.21%, 66.66%, and 96.86% of apoptotic cells were observed among those treated with AUY922-loaded micelles, whereas 48.75%, 64.34%, and 97.75% of apoptotic cells were observed among those treated with transferrin-targeted AUY922-loaded micelles for P5, P5-TMZ-R, and U87 brain cancer cells, respectively. In general, this result revealed that free AUY922 or nanoformulated AUY922 induces an apoptosis cascade to kill all tested brain cancer cells. Furthermore, to support the annexin v flow cytometry results, we conducted Western blotting to check the expression of certain apoptosis markers. As shown in Figure 5B, free AUY922 or nanoformulated AUY922 treatment-induced caspase-dependent PARP cleavage and the upregulation of p53 expression plays a key role in apoptosis of cancer cells. These results indicated that free AUY922 or nanoformulated AUY922 can kill P5, P5-TMZ-R, and U87 brain cancer cells through inducing apoptosis, and cleaved caspase-3 was the apoptotic executor. Western blot densitometry and uncropped blots are shown in the Supplementary Materials (Figure S7).

Moreover, cell cycle analysis was carried out using P5, P5-TMZ-R, and U87 cancer cells after treatment with free AUY922, NP-AUY922, and TF-NP-AUY922. As shown in Figure 5C and Figure S2, P5, P5-TMZ-R, and U87 brain cancer cells treated with free AUY922, NP-AUY922 and TF-NP-AUY922 showed a higher accumulation of cells in the G2M phase compared to that of the untreated P5, P5-TMZ-R, and U87 brain cancer cells. Several types of research have also reported the AUY922-mediated cell cycle arrest at G2M checkpoints [44].

### 3.5. Cellular Uptake Studies

**Figure 6:**
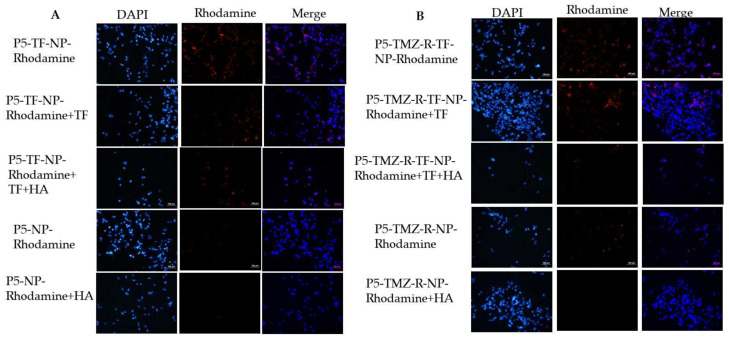
Cellular uptake of rhodamine B-encapsulated micelle and competitive inhibition assay using free transferrin, hyaluronic acid, or both using (**A**) P5 and (**B**) P5-TMZ-R cancer cells. Note: free transferrin and hyaluronic acid were incubated with the P5-and P5-TMZ-R brain cancer cells for the 1 h before treating with the rhodamine B-encapsulated micelle. The scale bar is 100 μm.

In drug delivery, the cellular uptake of NPs by the cells of interest is an essential step to obtain high therapeutic efficacy. Hence, understanding the cellular uptake and intracellular trafficking of NPs is crucial in the design of nanocarriers. Herein, the cellular uptake of the rhodamine B-loaded micelle was tested by fluorescence microscopy. As shown in Figure S3A,B, after 3 h of incubation with the rhodamine-loaded micelle, the red color fluorescence was observed in the cytoplasm of P5 and P5-TMZ-R brain cancer cells, which confirms the rhodamine B-loaded micelle was successfully taken up by cancer cells. Most interestingly, the fluoresce intensity of the transferrin-targeted rhodamine-loaded micelle was much higher than the untargeted rhodamine-encapsulated micelle for both cancer cell lines. This may be due to TF conjugation on the surface of micelle, which will enhance the cellular uptake of rhodamine, in addition to the HA intrinsic targeting capability. To further demonstrate the active tumor-targeting ability of transferrin and hyaluronic acid, competitive binding experiments were performed using free transferrin, free hyaluronic acid, or a combination of both. As shown in Figure 6A,B, after treatment with free transferrin and hyaluronic acid, the fluorescence intensity of rhodamine B, red color in the cytoplasm, was decreased. Most interestingly, the fluorescence intensity of rhodamine B was much more decreased in the presence of both free HA and free TF, which shows the synthesized micelle was taken up via transferrin or CD44 receptor, which is overexpressed on the surface of most cancer cells, including P5, P5-TMZ-R and U87 brain cancer cells (Figure S5). Similarly, flow cytometry results, shown in Figure S4, confirmed that TF-targeted rhodamine-loaded micelle uptake was more than the untargeted micelle. In addition, competitive inhibition was observed after incubation with free HA, free TF, and both free TF and HA. The percentage of rhodamine-loaded micelle uptake was decreased in both cell lines, as shown in the Supplementary Materials, which supports fluorescence microscopy results.

### 3.6. Free AUY922 and Nanoformulated AUY922 Inhibits HSP90 and Downregulates HSP90 Client Proteins in P5, P5-TMZ-R, and U87 Brain Cancer Cells

**Figure 7:**
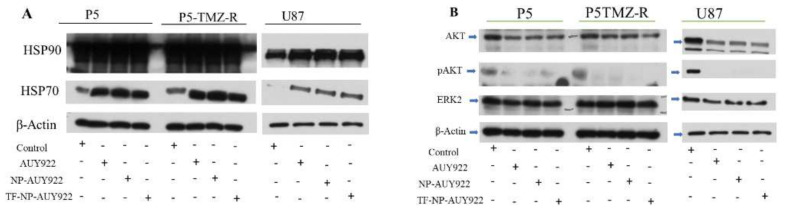
(**A**) Expression levels of HSP90, HSP70, and β-actin proteins (**B**) expression of AKT, pAKT, ERK2, and β-actin proteins in P5, P5-TMZ-R, and U87 brain cancer cells after treatment for 48 h using 100 nM free AUY922, NP-AUY922, and TF-NP-AUY922. In (**A**), the actin in the P5/P5-TMZ-R part of the experiment is identical as shown in Figure 5B, and the actin in the U87 part of the experiment in both (**A**,**B**) are the same, which was identical as shown in Figure 5B, because they are part of the same original blots probed with different antibodies of interest. The figures are re-organized as presented to better align with the data demonstrated in the Results section.

HSP90 plays a key role in normal and malignant cells by stabilizing chaperone proteins. Several research findings show that cancer cells overexpress HSP90 in comparison to the normal cell and play a great role in the stability of oncoproteins [45]. Hence, the inhibition of HSP90 is one of the important target sites in cancer research. Of this, AUY922 is a highly potent non-geldanamycin HSP90 inhibitor. To investigate HSP90 inhibition by free AUY922 or nanoformulated AUY922, Western blotting was performed using P5, P5-TMZ-R, and U87 brain cancer cells. As shown in Figure 7A, HSP70 induction was observed after treating all cell lines using free AUY922 and nanoformulated AUY922. This confirms AUY922 and nanoformulated AUY922 were successfully taken up by the cells and inhibited HSP90 since the upregulation of HSP70 shows the inhibition of HSP90 [46,47]. During HSP90 inhibition, heat shock factor 1 (HSF1) monomer dissociated, trimerized, and translocated to the nucleus to activate stress-responsive transcription including HSP70 [48]. To explore the detailed molecular action of free AUY922 or nanoformulated AUY922, we also assessed the expression pattern of HSP90 client proteins, such as AKT, pAKT, and ERK2. From Western blotting results (Figure 7B), all treatment groups, either with free AUY922 or nanoformulated AUY922, showed the expression of AKT, ERK2, and pAKT proteins were downregulated. This confirms that the inhibition of HSP90 using AUY922 is a crucial step to downregulate oncoproteins which have several functions, including promoting survival, proliferation and preventing apoptosis, resistance to anticancer agents, invasiveness, and metastasis [49]. Western blot densitometry and uncropped blots are shown in the Supplementary Materials (Figure S7).

Most interestingly, as revealed in the in vitro cytotoxicity studies such as MTT, colony or apoptosis assays, AUY922 or AUY922-loaded micelles tend to reverse TMZ resistance in brain cancers (i.e., P5-TMZ-R), which is accompanied by the inhibition of the AKT and ERK2 signaling pathways, as highlighted by decreased phosphorylated forms of AKT kinases, pAKT. Similar results have been reported for cisplatin resistance, which has been reversed after HSP90 inhibition in different cancer cells [50,51,52].

### 3.7. In Vivo and Ex-Vivo Biodistribution Studies

**Figure 8:**
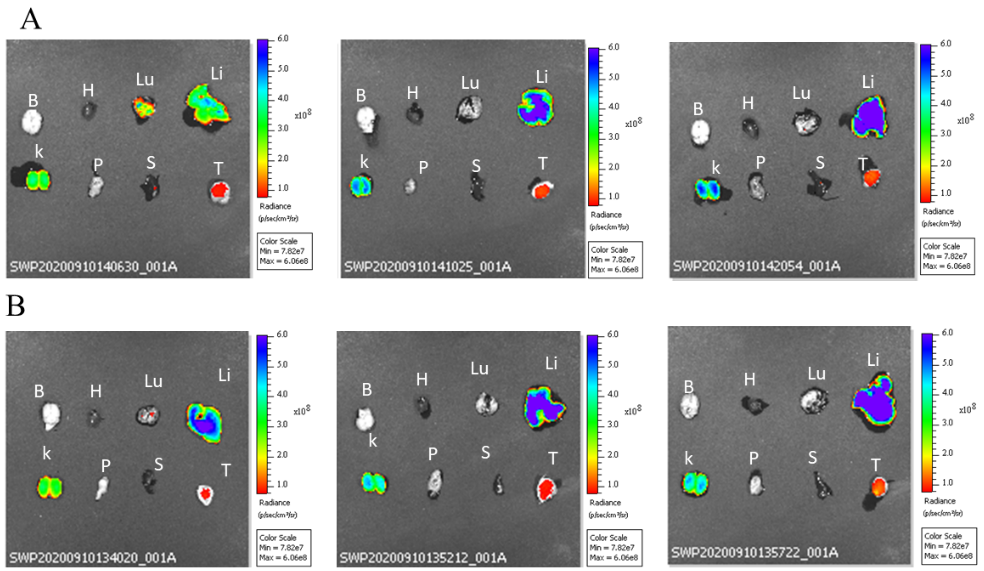
Ex vivo fluorescence image of selected major organs for (**A**) NP-cypate and (**B**) TF-NP-cypate (at cypate dosage: 1 mg/kg of mice). Note: brain (B), heart (H), lung (Lu), liver (Li), kidney (K), pancreas (P), spleen (S), and tumor (T).

Biodistribution studies of cypate-loaded micelles were evaluated on mice bearing U87 tumors and an in vivo biodistribution image was taken at different time intervals (i.e., 0, 1, 3, 6, and 24 h) using an IVIS spectrum in vivo imaging system. As shown in Figure S6A,B, a cypate-loaded micelle was biodistributed in the NOD-SCID mice bearing U87 tumors. The fluorescence intensity signals at each time interval vary, and high fluorescence intensity was observed at 1 and 3 h of post-injection, while fluorescence intensity was gradually decreased as the time increased and minimum fluorescence intensity was observed after 24 h of injection, which may be related to cypate degradation. In addition, ex vivo biodistribution studies were conducted by sacrificing a mouse after 24 h post-injection. As showed in Figure 8A,B, high fluorescence intensity was observed in the liver, kidney, and tumor tissue compared to the other organs. High fluorescence intensity in the liver and kidney may be related to cypate metabolism (i.e., the majority of drugs are metabolized in the liver) and excretion, which takes place in these two major organs, respectively.

### 3.8. In Vivo Therapeutic Studies

**Figure 9:**
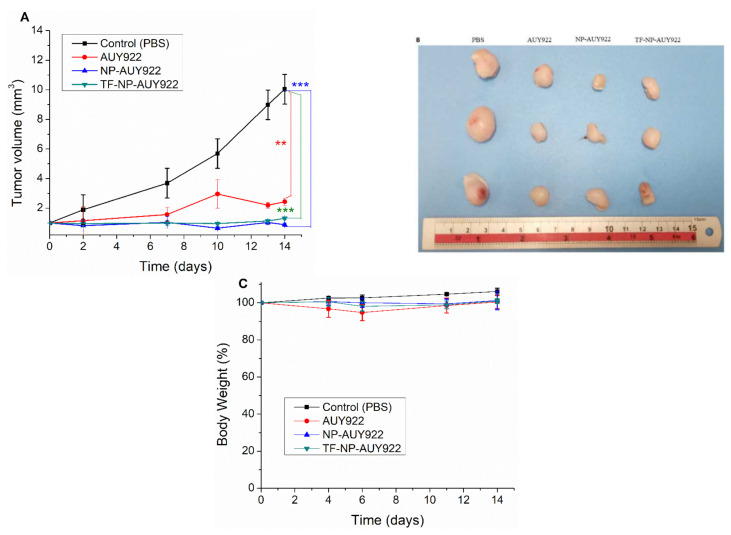
In vivo therapeutic efficacy of PBS (as a control), free AUY922, NP-AUY922, and TF-NP-AUY922 using U87 brain cancer-bearing NOD-SCID mice. The drugs were given on days 0, 3, 7, 10, and 13 (dosage: 20 mg/kg of mice for nanoformulated AUY922 and 40 mg/kg of mice for free AUY922). (**A**) Relative tumor volume, (**B**) selected images of the tumors collected on day 14, and (**C**) relative bodyweight of NOD-SCID mice. Data are presented as mean ± SD (*n* = 5). (** *p* < 0.01, *** *p* < 0.001). A one-tailed t-test was used for the p-value calculation for each control vs. treatment group.

The therapeutic efficacy of nanoformulated AUY922 and free AUY922 was investigated using NOD-SCID mice (five mice in each group) bearing U87 tumor cells. As depicted in Figure 9A, the PBS-treated groups (as the control) showed rapid tumor growth compared to that in mice treated with free AUY922 and nanoformulated AUY922. Most interestingly, nanoformulated AUY922 exhibited significantly better tumor growth inhibition at a lower concentration (20 mg/kg) than the free AUY922 (40 mg/kg). This could be due to the targeting capability of transferrin or hyaluronic acid which enhances AUY922 accumulation in the tumor regions to enhance the therapeutic efficacy by reducing tumor growth. The images of the tumors isolated on day 14 verified that mice treated with nanoformulated AUY922 had the smallest tumor size (Figure 9B) in comparison to the control group and free AUY922. Furthermore, no significant changes were observed in the percentage of body weight, as shown in Figure 9C, which shows synthesized micelle is biocompatible.

### 3.9. Organ Function Test and H&E Staining

**Figure 10:**
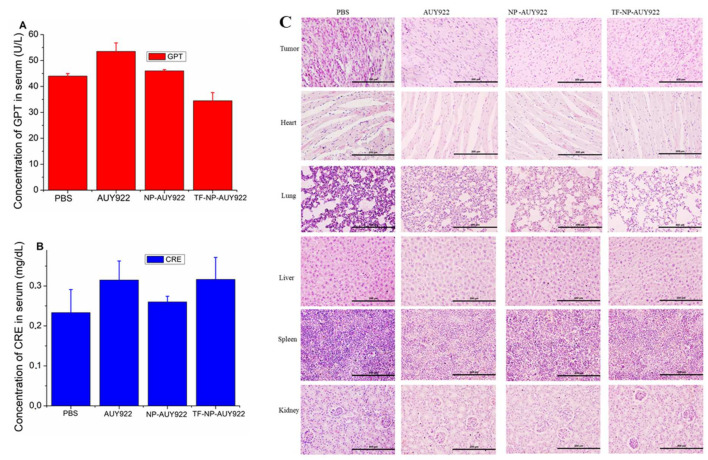
Toxicity evaluation of free AUY922, NP-AUY922, and TF-NP-AUY922 in comparison to control groups after 14 days of injections (**A**) Liver function test, (**B**) Kidney function test, (**C**) H&E staining. Scale bar represents 200 μm and magnification 20×.

Biochemical index analysis is one of the alternative ways to find out the biocompatibility of free drugs or nanoformulated drugs. Hence, to investigate the cytotoxicity of free AUY922 or nanoformulated AUY922, a blood sample was collected at the end of in vivo therapeutic study, and serum was separated to analyze the major biochemical markers for liver and kidney function tests. As shown in Figure 10A,B, there were no significant differences in the liver function test (GPT) and kidney function test (CRE) between the treatment group and control (PBS) group. Furthermore, the H&E staining method was used to evaluate any possible toxicity induced by the treatment. The results of H&E staining (Figure 10C) showed no inflammation or necrosis in the heart, lung, liver, spleen, and kidney for nanoformulated AUY922 or free AUY922 groups compared with PBS groups, indicating that the free AUY922 or nanoformulated AUY922 administration had no detectable toxicity in mice at the tested concentration. However, free AUY922 or nanoformulated AUY922 showed more tumor cell death in comparison to the control (PBS) groups.

## 4. Conclusions

In this study, a stable and biocompatible GSH-sensitive hyaluronic acid-poly (lactic-co-glycolic acid) (HA-SS-PLGA) was synthesized. Due to its amphiphilic nature, it forms a stable micelle in the aqueous medium to encapsulate hydrophobic AUY922. Drug release study showed higher drug release in the presence of 5 mM GSH than at physiological pH value. P5 and P5-TMZ-R cancer cells efficiently took up rhodamine B-encapsulated micelle as observed from fluorescence microscopy results. In vitro cytotoxicity results revealed that TF-NP-AUY922 shows higher cytotoxicity than the free AUY922 or NP-AUY922. In vivo and ex vivo biodistribution studies showed that the cypate-loaded micelle was accumulated in the tumor regions. Furthermore, nanoformulated AUY922 has significantly suppressed tumor growth in tumor-bearing NOD-SCID mice in comparison to the control groups. Overall, a synthesized micelle would be a promising carrier for AUY922 to enhance the therapeutic efficiency of brain cancer.

## Figures and Tables

**Table 1:** The IC50 value for P5, P5-TMZ-R, and U87 brain cancer cells after treated with free AUY922, NP-AUY922, and TF-NP-AUY922.

Cell Type	IC50 Value (nM)		
	Free AUY922	NP-AUY922	TF-NP-AUY922
P5	~37	~17	~17
P5-TMZ-R	~39	~17	~12
U87	~16	~14	~10

## Data Availability

Data is contained within the article or supplementary material.

## Supplementary Materials

The following are available online at https://www.mdpi.com/article/10.3390/cancers13102375/s1, Figure S1: Intensity ratio (I334/I328) from the pyrene emission spectra as a function of HA-SS-PLGA concentration (Log concentration), Figure S2: Cell cycle analysis of free AUY922, NP-AUY922 and TF-NP-AUY922 using P5 and P5-TMZ-resistant cancer cells after treating for 48 h (at 100 nM AUY922 concentration), Figure S3: Cellular uptake of rhodamine B-encapsulated micelle using fluorescence microscopy after incubating for the 3 h using P5 and P5-TMZ-R brain cancer cells, Figure S4: Cellular uptake of rhodamine B-encapsulated micelle using flow cytometry after 3 h of incubation. Competitive inhibition assay was carried out in the presence of free transferrin, hyaluronic acid, or both. Note: free transferrin and hyaluronic acid was incubated with the P5-and P5-TMZ-R brain cancer cells for the 1 h before treating with the rhodamine B-encapsulated micelle, Figure S5: Transferrin (CD71) and hyaluronic acid (CD44) receptor expression in P5, P5-TMZ-R, and U87 brain cancer cells, Figure S6: In vivo biodistribution study of (A) NP-cypate (B) TF-NP-cypate in U87 tumor-bearing NOD-SCID mice at 0, 1, 3, 6 and 24 h of post-injection, Figure S7: Western blot densitometry and uncropped blots, Table S1: The mean particle size, PDI, Zeta potential, DL (%) and EE (%) of empty and AUY922-loaded micelles at 25 °C. All expression ratio is carried out by dividing treated groups to control groups. Finally, all the expression ratio was normalized using β-actin.
