# Supplementary material for: Transferrin Modified GSH Sensitive Hyaluronic Acid Derivative Micelle to Deliver HSP90 Inhibitors to Enhance the Therapeutic Efficacy of Brain Cancers
Source: Cancers (Basel). 2021 May 14;13(10):2375. doi: 10.3390/cancers13102375 (PMC8156315; doi:10.3390/cancers13102375)
Supplement: Supplementary file 1 [file cancers-13-02375-s001.zip › cancers-1145157-supplementary.pdf]

cancers

Supplementary Materials

Transferrin Modified GSH Sensitive Hyaluronic Acid Derivative Micelle to Deliver HSP90 Inhibitors to Enhance the Therapeutic Efficacy of Brain Cancers

Tilahun Ayane Debele, Ping-Ching Wu, Yu-Feng Wei, Jian-Ying Chuang, Kwang-Yu Chang, Jui-Hung Tsai and Wen-Pin Su

Cell Preparation

The human primary GBM cell line P5 was obtained according to a protocol approved by the Taipei Medical University (TMU) Internal review board (approval no. 201006011) [1]. The P5-TMZ-R was resistant cell line to temozolomide (TMZ), derived from P5. Both P5 and P5-TMZ-R brain cancer cells were maintained and cultured according to their previous reports [2]. Briefly, cells were incubated in Dulbecco's modified Eagle's medium (Thermo Fisher Scientific, Waltham, MA, USA) with 10% fetal bovine serum (Thermo Fisher Scientific), 1% antibiotic-antimycotic solution (Corning). P5-TMZ-R cells were maintained in the same culture medium containing 50μM TMZ (Sigma-Aldrich, St. Louis, MO, USA). The human cell line U87 was purchased from American type culture collection (Manassas, VA, USA) and incubated in Eagle's Minimum Essential Medium (EMEM) with 10% fetal bovine serum (Thermo Fisher Scientific), 1% non-essential amino acids (GeneDirex, Inc.,), 1% Sodium pyruvate (GeneDirex, Inc.,) and 1% antibiotic-antimycotic solution (Corning). All cells were incubated in a humid atmosphere at 37°C and 5% CO_2.

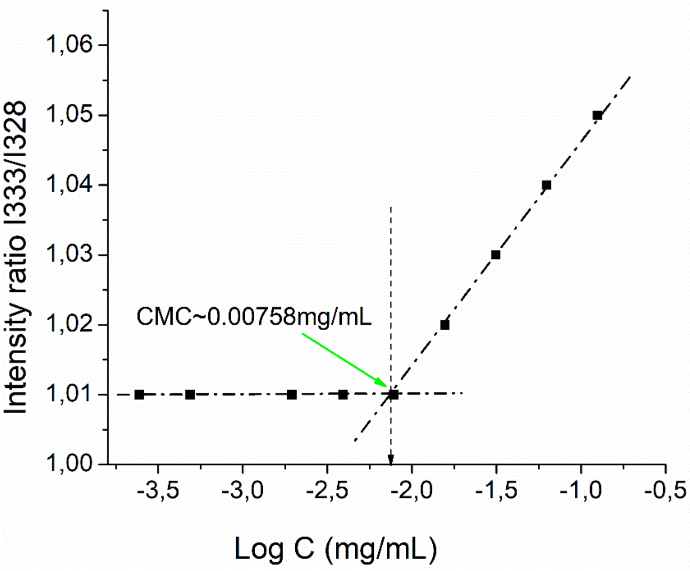

Figure S1. Intensity ratio (I334/I328) from the pyrene emission spectra as a function of HA-SS-PLGA concentration (Log concentration).

Table S1. The mean particle size, PDI, Zeta potential, DL (%) and EE (%) of empty and AUY922-loaded micelles at 25 ℃.

s/No	Sample Name	Size (nm)	PDI	Zeta (mv)	DL%	EE%

Copyright: © 2021 by the authors. Licensee MDPI, Basel, Switzerland. This article is an open access article distributed under the terms and conditions of the Creative Commons Attribution (CC BY) license (http://creativecommons.org/licenses/by/4.0/).

1	NP-AUY922	227.27 ± 5.10	0.18 ± 0.06	−36.23 ± 1.97	10.37	87.0 ± 0.58
2	NP	205.33 ± 5.78	0.11 ± 0.09	−35.9 ± 2.92	NA	NA
3	NP-AUY922 + 5mM GSH	1290.97 ± 39.4	0.59 ± 0.08	−2.5 ± 1.47	NA	NA
4	NP + 5mM GSH	466.9 ± 41.57	0.59 ± 0.33	−1.83 ± 2.05	NA	NA
5	NP-AUY922 + 10% FBS	226.73 ± 2.82	0.21 ± 0.02	-3.2 ± 0.1	NA	NA
6	NP + 10% FBS	208.53 ± 2.34	0.11 ± 0.02	−4.1 ± 0.78	NA	NA

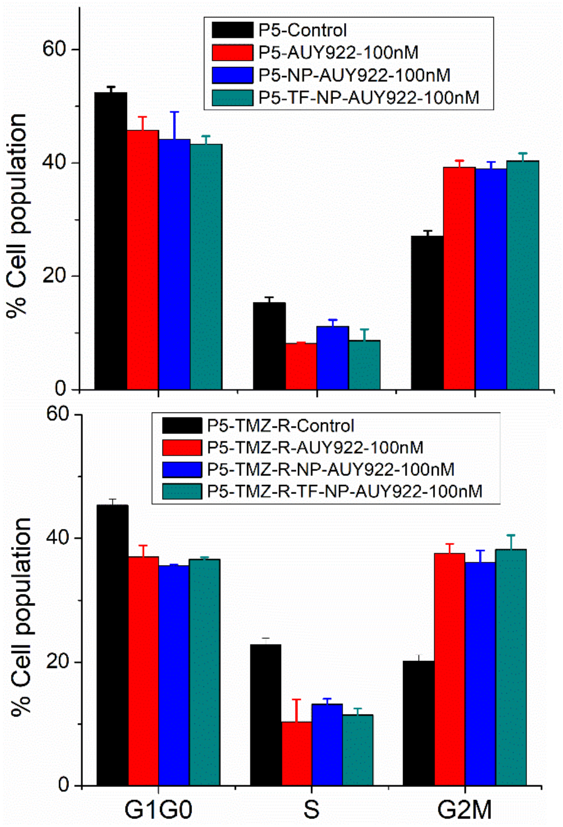

Figure S2. Cell cycle analysis of free AUY922, NP-AUY922, and TF-NP-AUY922 using P5 and P5-TMZ-resistant cancer cells after treating for 48h (at 10 nM AUY922 concentration).

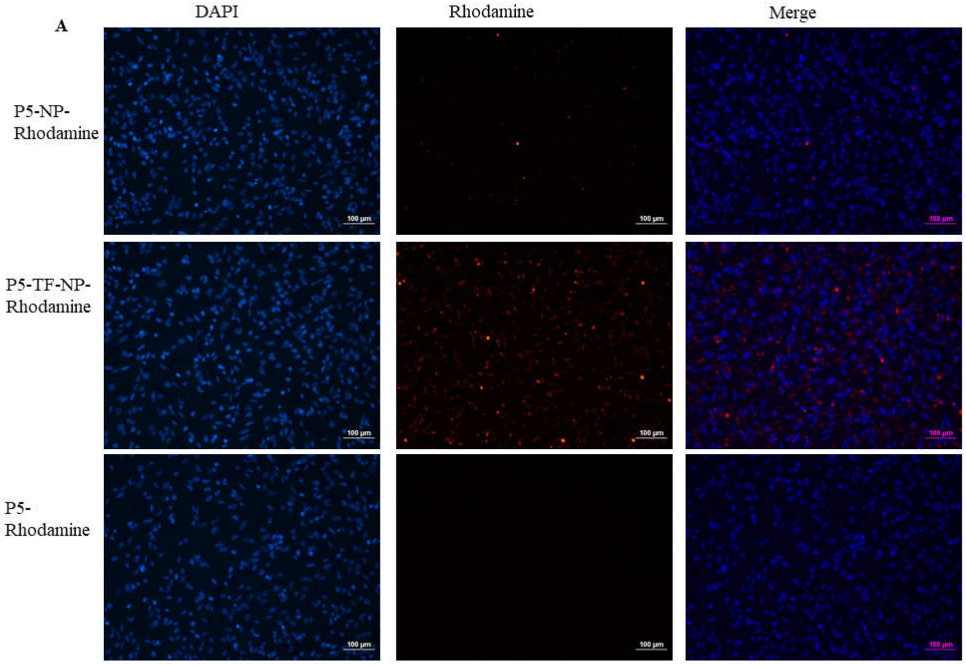

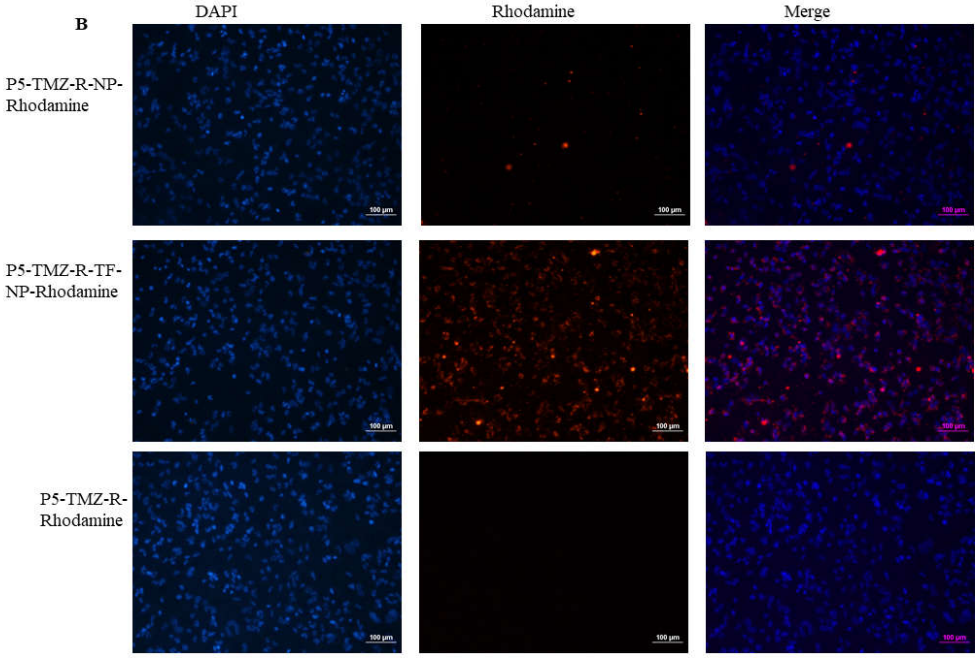

Figure S3. Cellular uptake of Rhodamine B encapsulated micelle using fluorescence microscopy after incubating for the 3h using P5 and P5-TMZ-R brain cancer cells.

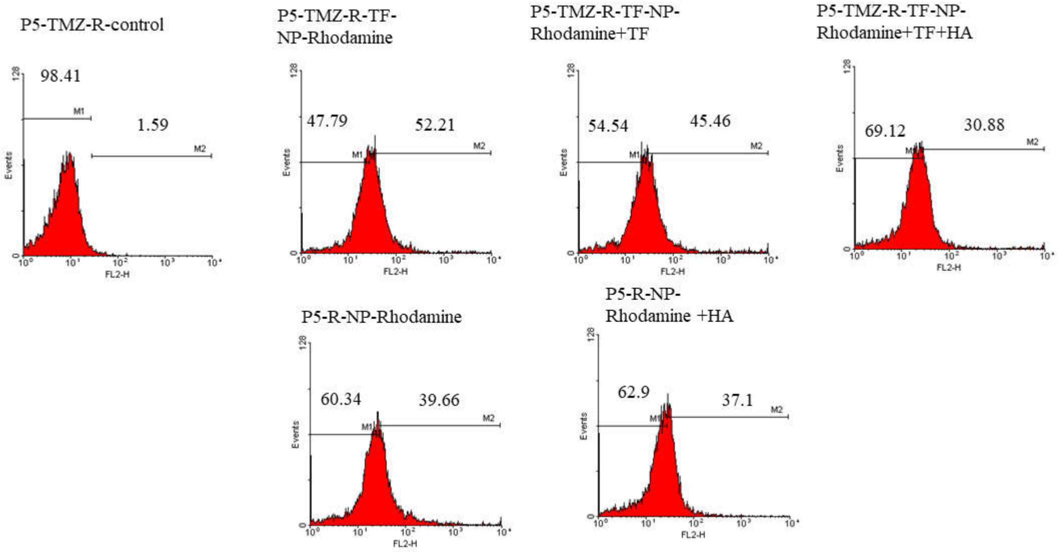

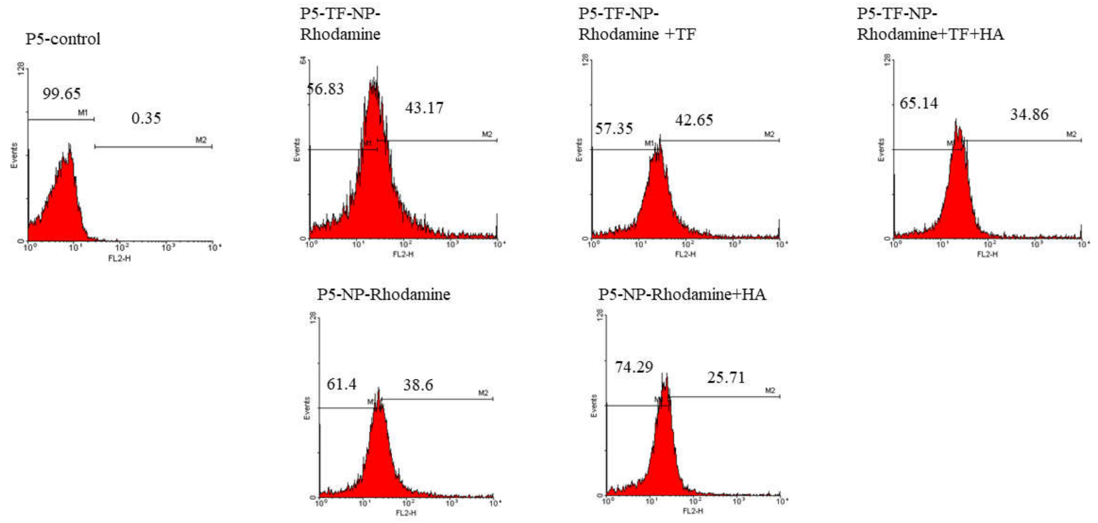

Figure S4. Cellular up take of Rhodamine B encapsulated micelle using flow cytometry after 3h of incubation. Competitive inhibition assay was done in the presence of free Transferrin, hyaluronic acid, or both. Note: - free Transferrin and hyaluronic acid were incubated with the P5-and P5-TMZ-R brain cancer cells for the 1h before treating with the Rhodamine B encapsulated micelle.

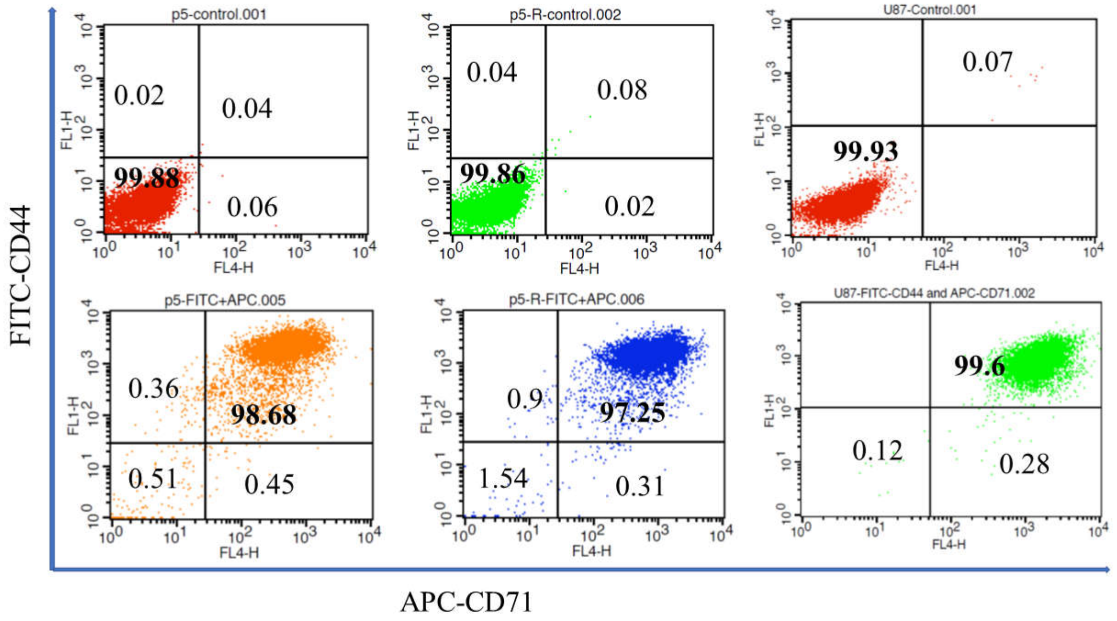

Figure S5. Transferrin (CD71) and Hyaluronic acid (CD44) receptor expression in P5, P5-TMZ-R, and U87 brain cancer cells.

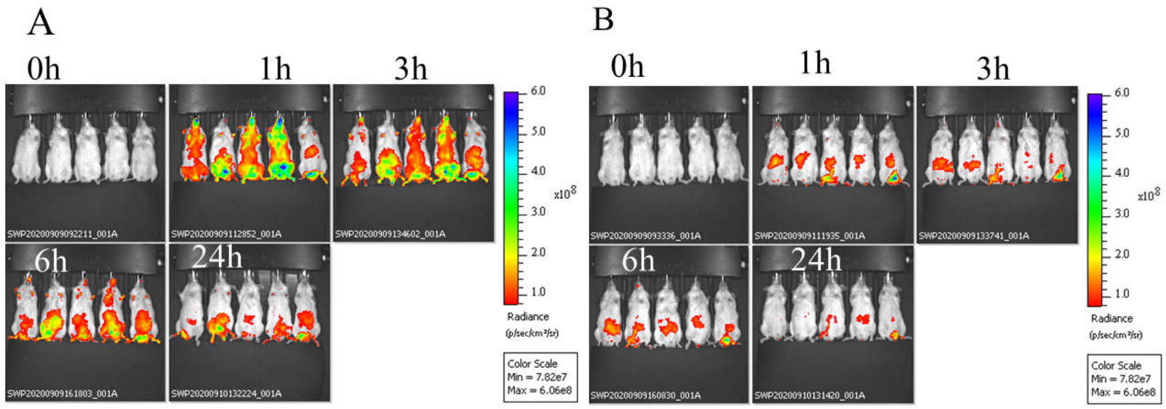

Figure S6. In vivo biodistribution study of (**A**) NP-cypate (**B**) TF-NP-cypate in U87 tumor-bearing NOD-SCID mice at 0, 1, 3, 6, and 24 h of post-injection.

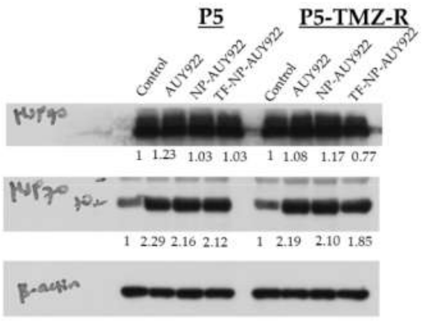

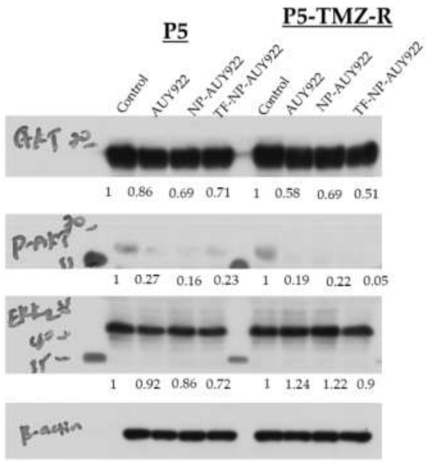

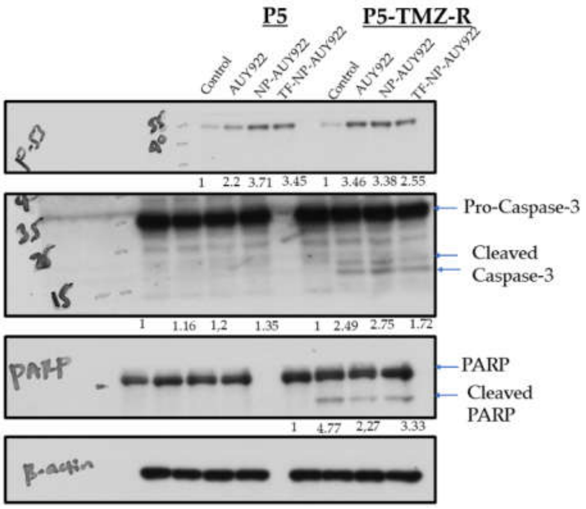

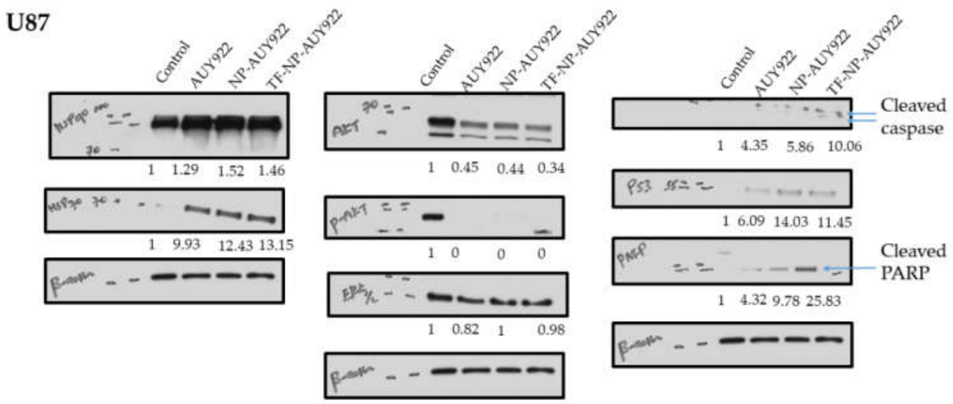

Figure S7. Western blot densitometry & uncropped blots. All expression ratio is done by dividing treated groups to control groups. Finally, all the expression ratio was normalized using β-actin.

Reference

1. Chang, K.-Y.; Huang, C.-T.; Hsu, T.-I.; Hsu, C.-C.; Liu, J.-J.; Chuang, C.-K.; Hung, J.-J.; Chang, W.-C.; Tsai, K.K.; Chuang, J.-Y. Stress stimuli induce cancer-stemness gene expression via Sp1 activation leading to therapeutic resistance in glioblastoma. *Biochem. Biophys. Res. Commun.* **2017**, *493*, 14–19,
2. Lo, W.-L.; Hsu, T.-I.; Yang, W.-B.; Kao, T.-J.; Wu, M.-H.; Huang, Y.-N.; Yeh, S.-H.; Chuang, J.-Y. Betulinic Acid-Mediated Tuning of PERK/CHOP Signaling by Sp1 Inhibition as a Novel Therapeutic Strategy for Glioblastoma. *Cancers* **2020**, *12*, 981, doi:10.3390/cancers12040981.
